# Shedding light on the role of CX3CR1 in the pathogenesis of schizophrenia

**DOI:** 10.1007/s43440-021-00269-5

**Published:** 2021-05-22

**Authors:** Katarzyna Chamera, Magdalena Szuster-Głuszczak, Agnieszka Basta-Kaim

**Affiliations:** Laboratory of Immunoendocrinology, Department of Experimental Neuroendocrinology, Maj Institute of Pharmacology, Polish Academy of Sciences, 12 Smętna St., 31-343 Kraków, Poland

**Keywords:** Schizophrenia, CX3CL1, CX3CR1

## Abstract

Schizophrenia has a complex and heterogeneous molecular and clinical picture. Over the years of research on this disease, many factors have been suggested to contribute to its pathogenesis. Recently, the inflammatory processes have gained particular interest in the context of schizophrenia due to the increasing evidence from epidemiological, clinical and experimental studies. Within the immunological component, special attention has been brought to chemokines and their receptors. Among them, CX3C chemokine receptor 1 (CX3CR1), which belongs to the family of seven-transmembrane G protein-coupled receptors, and its cognate ligand (CX3CL1) constitute a unique system in the central nervous system. In the view of regulation of the brain homeostasis through immune response, as well as control of microglia reactivity, the CX3CL1–CX3CR1 system may represent an attractive target for further research and schizophrenia treatment. In the review, we described the general characteristics of the CX3CL1–CX3CR1 axis and the involvement of this signaling pathway in the physiological processes whose disruptions are reported to participate in mechanisms underlying schizophrenia. Furthermore, based on the available clinical and experimental data, we presented a guide to understanding the implication of the CX3CL1–CX3CR1 dysfunctions in the course of schizophrenia.

## Introduction

Schizophrenia is a chronic and severe mental illness, ranked among the leading causes of disability worldwide in recent years [1–3]. Despite a relatively low prevalence, the condition is one of the major contributors to the global burden of disease [2, 3]. The onset of that disorder usually appears in late adolescence or early adulthood [4]. The diagnosis of schizophrenia is based on clinical criteria that consider varied symptomatology, generally categorized into three groups: positive symptoms including hallucinations, delusions or conceptual disorganization; negative symptoms consisting of blunted or loss of affect and conative functions, avolition or apathy; and cognitive deficits referring to impairment of various types of memory and difficulty processing and using information [5–7]. Although the causes of schizophrenia remain unclear, the heterogeneous nature of the condition implies the contribution of multiple aetiological factors. The reports have suggested that the development of this illness may result among others from genetics [8–10], altered brain connectivity [11–15], abnormalities in neurotransmission systems [16–21] and/or environmental factors, including childhood trauma [22, 23], maternal stress [24] and infections during pregnancy [25–27], obstetric complications [28] as well as prenatal malnutrition [29]. The interplay between some of these factors, for example, gene–environment interactions [30–33] with an increased focus on epigenetic regulation [34–36], has been also proposed as the basis of this disorder. Recently, even though diversity in research data emerges [37–39], multiple studies have strongly supported the role of an inflammatory component in the pathogenesis of schizophrenia [40–42]. It has been shown that patients with this disease suffer from disturbances in the expression of cytokines and chemokines with inter alia the affected levels of interleukin-1β (IL-1β), IL-2, IL-1 receptor antagonist (IL-1RA), and elevated production of IL-6, IL-8, tumour necrosis factor α (TNF-α), monocyte chemoattractant protein-1 (MCP-1) and C–C motif chemokine ligand 5 (CCL5 or RANTES) in blood or cerebrospinal fluid [43–48]. Additionally, polymorphisms in cytokine genes such as *IL-2*, *IL-6*, *IL-10* and *TNF-α* are likely to be a risk factor for this disease [49–51]. Some postmortem studies have found the presence of activated microglia and changes in the levels of cytokines, chemokines and microglial markers [e.g., major histocompatibility complex class I (MHCI), MHCII, IL-1β, IL-6, IL-8] in brain tissues [39]. In the central nervous system (CNS), microglia are the main immunocompetent cells and primary reservoirs of inflammatory factors [52]. Even though microglia constitute only about 10% of the total brain cells [53], they respond rapidly to even minor pathological changes in the CNS and may contribute directly to brain homeostasis. Therefore, interest in the role of critical molecules modulating functions of microglia has been prompted in the context of mechanisms underlying schizophrenia. Among them, chemokines, in particular, have gained special attention with recent evidence suggesting the importance of the CX3CL1–CX3CR1 axis to this condition.

## The general characteristic of the CX3CL1–CX3CR1 system

**Fig. 1:**
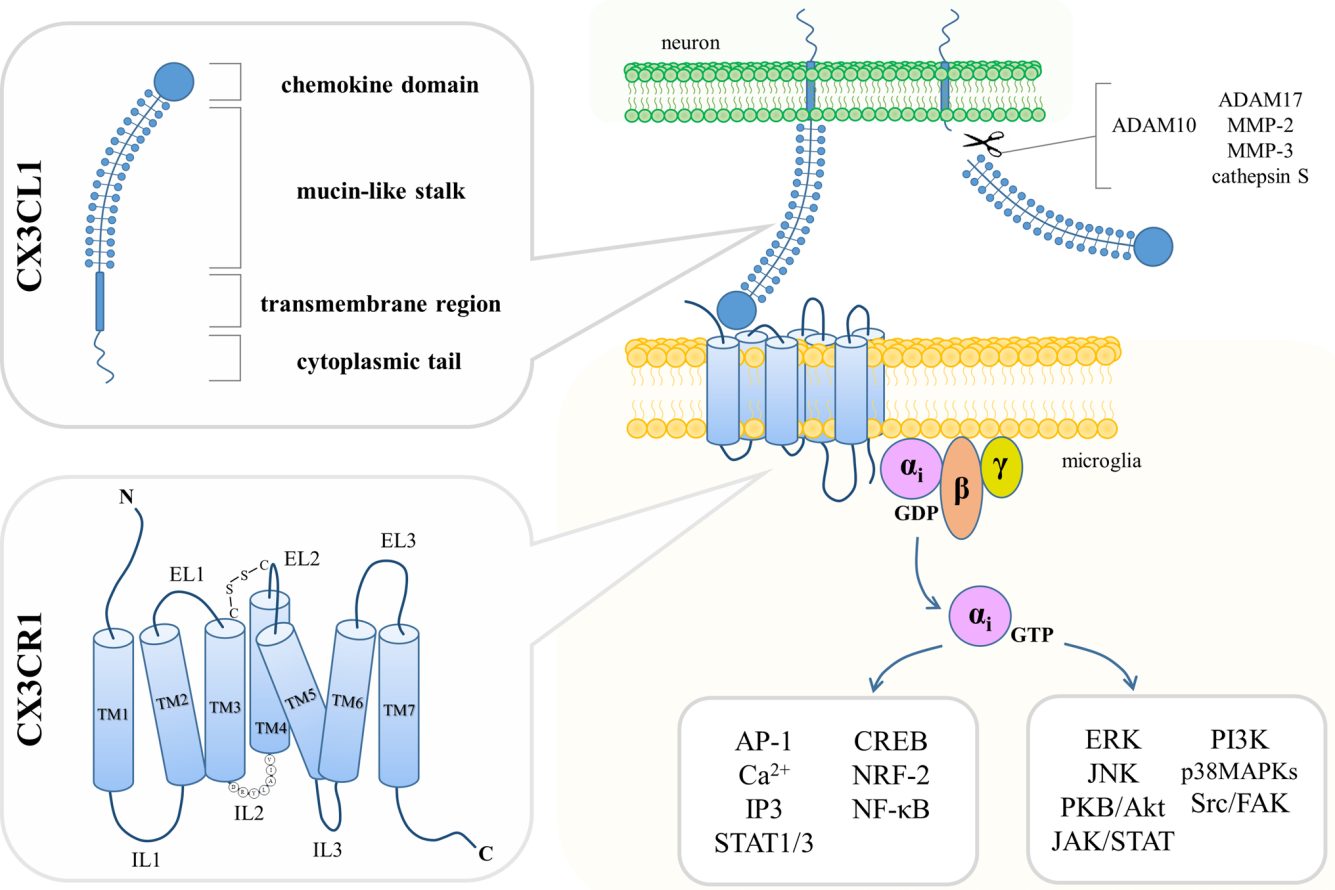
Scheme illustrating the structure, localization and signaling pathways affected by the CX3CL1–CX3CR1 axis. CX3CL1, produced mostly by neurons, is a membrane-bound molecule with a chemokine domain, mucin-like stalk, transmembrane region and cytoplasmic tail. Cleavage of CX3CL1 is mediated under physiological or pathological conditions by ADAM10 or ADAM17, MMP-2, MMP-3 and cathepsin S, respectively. Binding CX3CL1 to CX3CR1, which is a seven-transmembrane domain G_*i*_ protein-coupled receptor expressed primarily on microglia, results in an intracellular transmission engaging multiple signaling pathways. *TM* transmembrane domain, *EL* extracellular loop, *IL* intracellular loop

C-X3-C motif chemokine ligand 1 (CX3CL1) was firstly described in 1997 under the name “fractalkine” in humans [54] and simultaneously as “neurotactin” in mice [55]. This molecule differs notably from other classes of chemokines in terms of structure (Fig. 1). CX3CL1 is synthesized as an intracellular precursor (50–75 kDa) that undergoes rapid maturation processes to yield mature glycoprotein (95–100 kDa) transported to the cell surface [56, 57]. The full-length CX3CL1 is encoded by a 395–397-amino-acid chain and contains a chemokine domain, mucin-like stalk, transmembrane region and a cytoplasmic tail [54, 58, 59]. Due to the specific arrangement of two cysteine residues near the amino terminus divided from each other by three amino acids, it was assigned to a separate type of chemokines (δ subfamily) and it is the only known representative of the CX3C class so far [54, 55]. CX3CL1 appears in two forms: soluble (sCX3CL1) and membrane-bound (mCX3CL1) [60], which recently have been suggested to display differential activities within the CNS [61]. Under physiological conditions, the cleavage of sCX3CL1 is primarily carried out by a disintegrin and metalloproteinase domain-containing protein 10 (ADAM10) [62], while in the case of induction with a stress factor—by the TNF-α converting enzyme (TACE or ADAM17) [56, 63], matrix metalloprotease-2 (MMP-2) [64] and MMP-3 [65] or cathepsin S [66, 67]. It should be noted that there are some inconsistencies in the observed molecular weight of the secreted chemokine, possibly due to multiple forms of sCX3CL1 generated by the shedding from the cell surface at alternative sites [62, 68, 69]. Several reports have also shown that the *CX3CL1* gene is polymorphic and its genetic variants may be related to HIV infection [70], postoperative chronic pain [71], coronary artery disease [72, 73] and carotid intima-media thickness [74] as well as a reduced risk of major depression [75]. CX3CL1 is vastly distributed throughout the body with the predominant expression in the brain [76] and to a lesser extent in the heart, kidney, lung and uterus [55]. In the CNS, the distribution of this chemokine varies between regions [77]. The highest protein levels of CX3CL1 were detected in the amygdala, cerebral cortex (particularly in layers II, III, V and VI), hippocampus (most intensely in CA1 field), basal ganglia and olfactory bulb. Other brain structures such as the hypothalamus and brainstem showed a scattered and scant presence of CX3CL1. Concerning the gene expression of this chemokine, it corresponds with protein localization as the uppermost mRNA levels were measured in the hippocampus, cerebral cortex and striatum [77]. On the cellular level, the main sources of CX3CL1 are neurons [78] along with dendritic cells [79], endothelial cells [54, 80], epithelial cells [80, 81], fibroblasts [82], macrophages [83] and smooth muscle cells [84]. Within the brain, it was suggested that also astrocytes produce CX3CL1, however, at lower levels than neurons [85–87] or as a result of inflammatory stimulation, in an example with TNF-α and interferon γ (IFN-γ) [88].

CX3CL1 interacts with only one known receptor (Fig. 1). It was first described by the name RBS11 in rats [89] and later as V28 in humans [90]. However, since it has represented the first receptor for CX3CL1, it was accordingly designated as CX3C chemokine receptor 1 (CX3CR1) [91]. CX3CR1 is a seven-transmembrane domain G_*i*_ protein-coupled receptor (GPCR) and belongs to the A class, which includes rhodopsin-like receptors [91]. CX3CR1 (40 kDa) is composed of 355 amino acid residues forming an extracellular N-terminus, alternately arranged α-helical domains (TM1–TM7), intracellular (IL1–IL3) and extracellular (EL1–EL3) loops, and an intracellular C-terminus [92]. IL2 contains a DRY (also called DRYLAIV) motif, which is crucial for G protein interactions and signal transduction by the receptor [90, 91]. The research data have shown the presence of the receptor gene’s single-nucleotide polymorphisms (SNPs) resulting in two functional variants (V249I and T280M) [93], which to varying degrees have been associated with age-related macular degeneration [94], AIDS [95], amyotrophic lateral sclerosis [96, 97], coronary artery disease [98], Crohn’s disease [99], multiple sclerosis [100] and obesity [101]. Additionally, these SNPs may affect arterial blood volume in the precuneus, left posterior parietal cortex and left posterior cingulate cortex, structures with observed abnormalities in schizophrenia, bipolar disorder, autism and Alzheimer’s disease [102]. Recently, *CX3CR1* V249I polymorphism has been also suggested as a factor that improved overall and progression-free survival in low-grade gliomas [103]. Regarding the expression, CX3CR1 is present on microglia [104, 105], dendritic cells [106], mast cells [107], monocytes [108, 109], macrophages [108], natural killer cells [91, 110], neutrophils [108], T lymphocytes [108] and thrombocytes [111].

Binding the ligand to CX3CR1 results in an intracellular transmission mediated by several second messengers and transcription factors, including for instance activator protein 1 (AP-1) [112], Ca^2+^ [113, 114], cAMP response element-binding protein (CREB) [115], inositol 1,4,5-trisphosphate (IP3) [114], nuclear factor erythroid-derived 2-like 2 (NRF-2) [116], nuclear factor kappa-light-chain-enhancer of activated B cells (NF-κB) [112, 115, 117, 118] as well as signal transducers and activators of transcription 1/3 (STAT1/3) [112, 119]. The signal transduction by the receptor affects pathways engaging protein kinase B (PKB or Akt) [120–122], extracellular signal-regulated kinase (ERK) [118, 120, 123], Janus kinase (JAK)/STAT [112, 119, 124], c-Jun N-terminal kinases (JNK) [118, 121], p38 mitogen-activated protein kinases (p38MAPKs) [112, 115], phosphoinositide-3-kinase (PI3K) [120, 125] and steroid receptor coactivator/focal adhesion kinase (Src/FAK) [126, 127] (Fig. 1). Regulation of these signaling pathways underlines the reported in literature roles of the CX3CL1–CX3CR1 axis both in physiological and pathological processes within the organism.

## The involvement of the CX3CL1–CX3CR1 dyad in the brain physiology

**Fig. 2:**
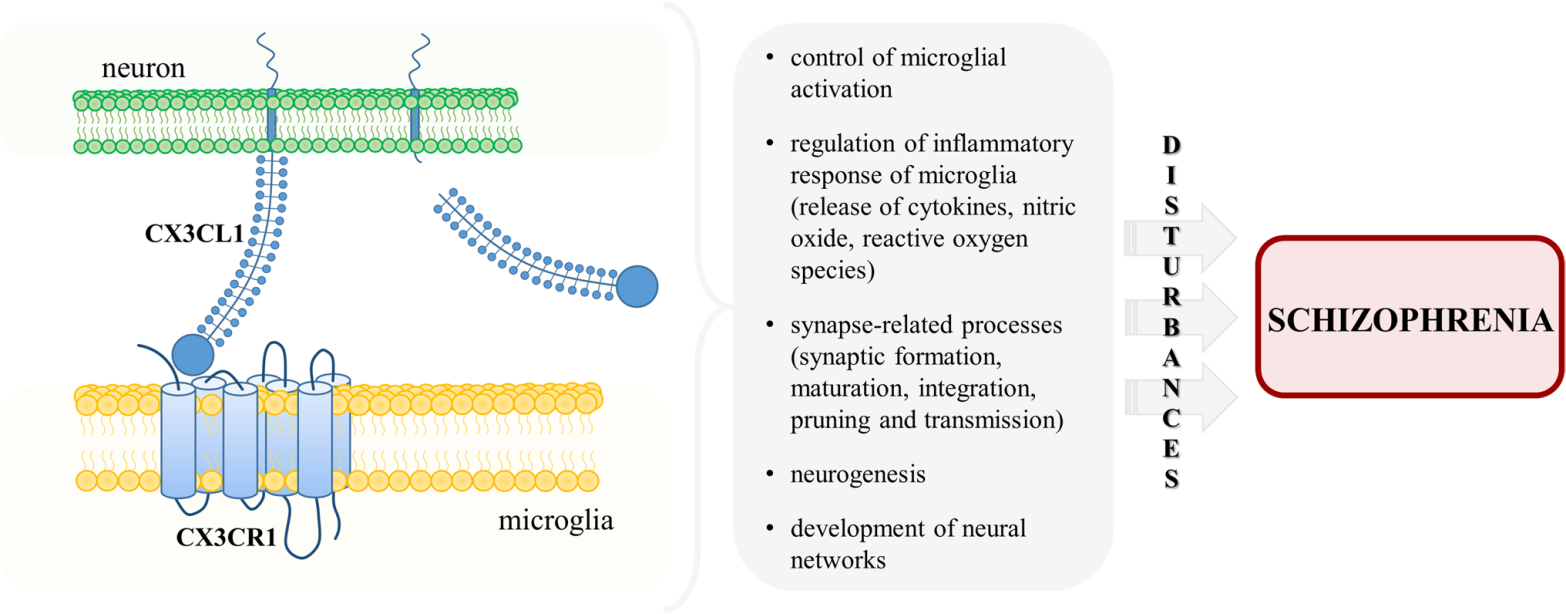
The role of the CX3CL1–CX3CR1 signaling pathway in the pathology of schizophrenia. In physiological conditions, the interaction of CX3CL1 with CX3CR1 is essential for the regulation of multiple processes in the brain. The disturbances within this axis and subsequent disruptions within these mechanisms implicate the CX3CL1–CX3CR1 dyad in schizophrenia

The participation of CX3CR1 activation by its ligand in homeostatic conditions has been already addressed in impressive details in a few excellent articles, both experimental and review [128–133]. We invite the reader to get acquainted with these publications, and therefore in this chapter, we will briefly present only reports that are essential for understanding further data showing the CX3CL1–CX3CR1 system in the context of schizophrenia (Fig. 2).

The major role of the CX3CL1–CX3CR1 axis in the CNS covers control of the activation and functioning of microglia. For the first time, the phenomenon has been supported by the in vitro experiments, in which stimulation with the ligand triggered induction of Ca^2+^ mobilization, activation of MAPK and Akt, strong migratory activity and the reorganization of the actin cytoskeleton of these cells [104]. Additionally, Lyons et al. [134] described the decrease in CX3CL1 level in the hippocampus of aged rats that was accompanied by an increase of microglial activation. Treatment of those animals with the ligand diminished the activation, proving that CX3CL1 is required to maintain the cells in a quiescent state [134]. In line with this function, it has been shown that the interaction of CX3CL1 with CX3CR1 participates in the regulation of the inflammatory response of microglia, which includes a release of cytokines, nitric oxide and reactive oxygen species [122, 135–137]. The impairment of these processes, leading to prolonged microglial activation and neuroinflammation, has been indicated as part of schizophrenia pathology [138, 139].

It is widely recognized that the CX3CL1–CX3CR1 pair takes part in synapse-related processes, including synaptic formation, maturation, integration, pruning and transmission. The evidence has been provided by many data, including the research on *Cx3cr1*-deficient mice. In the article by Paolicelli et al. [140], these animals were characterized by transiently reduced microglia numbers in the developing brain and delayed synaptic pruning. The deficiency in this process resulted in an excess of dendritic spines and immature synapses and was associated with the persistence of electrophysiological and pharmacological indicators of immature brain circuitry [140]. Rogers et al. [141] demonstrated that mice lacking the *Cx3cr1* gene displayed a significant decrease in hippocampal neurogenesis, impaired synaptic plasticity and up-regulated level of pro-inflammatory IL-1β, followed by the behavioral changes (precisely, disrupted motor learning, associative and spatial memory). As presented by Bolós et al. [142], the depletion of the receptor caused the deficient synaptic integration of adult-born granule neurons in the hippocampal dentate gyrus, both at the afferent (a decreased number of dendritic spines) and efferent (a reduced area of axonal terminals) level. Other research revealed that *Cx3cr1*−/− knockouts exhibited alterations in postnatal functional maturation of thalamocortical synapses [143]. The above-mentioned data are particularly important in the context of multiple synapse pathologies (e.g., reduction in spine density, enrichment of rare disruptive variations in synaptic genes and increased synaptic pruning) observed in the brains of patients with schizophrenia [144, 145].

Synaptic remodeling and plasticity contribute to the development of neural networks [146–148]. Therefore, it seems natural that the CX3CL1–CX3CR1 dyad is engaged in the formation of these circuits within the brain. This subject was reviewed extensively in the article by Paolicelli et al. [129], where the authors collected convincing data implicating the interaction of CX3CL1 with its receptor in the formation and reconstruction of neural connectivity. The research findings have shown that anomalous circuitry is one of the hallmarks of schizophrenia as the changes in neural networks of, inter alia, the prefrontal cortex and hippocampal formation of the individuals with the illness have been found [149–153].

## The implication of the CX3CL1–CX3CR1 signaling pathway in schizophrenia

### Clinical data

To date, only a few studies have evaluated the expression of CX3CL1 and CX3CR1 in patients with schizophrenia (Table 1). As reported by Bergon et al. [154], meta-analyses of microarray data from postmortem brain and blood samples highlighted down-regulation of *CX3CR1* mRNA levels in the subjects affected by this condition. The finding was further confirmed by RT-qPCR examination in the peripheral blood mononuclear cells (PBMCs) obtained from the suffering from schizophrenia. The dysregulation of the gene expression in PBMCs was independent of confounding variables (including tobacco smoking, age, gender or antipsychotic medication) and correlated with a depression–anxiety phenotype [154]. Comparable results were presented by Fries et al. [155] whose genome-wide research revealed the diminished *CX3CR1* level in PBMCs from veterans with a diagnosis of schizophrenia. Similarly, the datasets-integrated analysis showed that the *CX3CR1* expression was decreased in the hippocampi of individuals with this illness [156]. Another evidence for this phenomenon was delivered by Gandal et al. [157] whose multifaceted and complex microarray study revealed a robust reduction in both *CX3CL1* and *CX3CR1* levels in postmortem cortical samples from patients with schizophrenia. The authors confirmed and expanded these findings in further examinations applying large-scale RNA-sequencing-based quantifications that integrated genetic and genomic data from numerous well-curated, high-quality postmortem brain specimens from individuals with the disease and controls [158]. The analyses of a transcriptomic organization at the levels of a gene, isoform, local splicing and gene networks indicated down-regulation in differential expression of *CX3CL1* and *CX3CR1* as well as the presence of variously expressed isoforms [158]. Possibly such changes regarding genetic variants may exhibit distinct biological effects and consequently result in heterogeneity in pathology progression or symptom manifestation in schizophrenia. In parallel to these observations, it was shown that the rare variant (Ala55Thr) in the *CX3CR1* gene contributes to the increased risk of this condition [159]. The researchers proved that the mutation could destabilize the conformation of the receptor by weakening the hydrophobic interaction between TM1 and helix 8 in the structure of *CX3CR1*. Consequently, the Ala55Thr variant affected the gene interplay with a G protein and resulted in inhibition of the CX3CL1–CX3CR1 signaling. Complementary experiments on HEK293 cells transfected with Ala55Thr-expressing vector demonstrated a reduction in Akt phosphorylation-mediated signaling upon CX3CL1 treatment [159]. A characterization of microglia-like cells derived from patients with schizophrenia demonstrated the presence of two unique inflammatory phenotypes within these cells [160]. It is noteworthy that one out of the significantly abundant clusters was distinguished by higher expression of *CX3CR1*. In another study, Zhang et al. [161] showed that *CX3CR1* transcript level was increased in the anterior cingulate cortex of suicide completers with schizophrenia when compared to the subjects affected by this condition who died of other causes. However, when the investigated cohort was analysed holistically and compared to controls, the mRNA expression of the receptor was unchanged in this brain structure (as well as in the dorsal lateral prefrontal cortex). These postmortem data emphasized the importance of accurate characteristic of examined groups in terms of symptoms of the disease and anamneses. Nonetheless, the latest articles implicate that the disruption in the CX3CL1–CX3CR1 signaling in schizophrenia may be limited to a shift in the ligand production. Hill et al. [162] described diminished protein release of CX3CL1 with no change in the level of CX3CR1 in the postmortem dorsolateral prefrontal cortex from individuals with this disorder. The decline in the expression of the ligand was not accompanied by the difference in ADAM10 production, suggesting that the lower level of CX3CL1 was not caused by the altered cleavage conducted by this sheddase. Additional analysis of the samples from patients with schizophrenia revealed a subtle but significant negative correlation between CX3CL1 protein level and lifelong antipsychotic dose. This association implies the possibility that chronic medication with antipsychotics may contribute to the reduced production of this chemokine [162]. In the same study, no discrepancies between control subjects and those affected by schizophrenia were found in terms of the transcript expression of neither *CX3CL1* nor *CX3CR1* in the orbitofrontal cortex. The observations regarding the protein and mRNA levels of the CX3CL1–CX3CR1 dyad were unrelated to such contributory factors as body mass index, serum C-reactive protein release, alcohol consumption, prescribed antidepressants or mood stabilizers, death by suicide and a subtype of schizophrenia (undifferentiated or paranoid). As noted by the authors, sex had an effect only on the CX3CL1 production in the control group as males were characterized by higher levels of the ligand than females [162].

**Table 1:** Summary of alterations in the CX3CL1–CX3CR1 axis protein levels and mRNA expression reported in the studies in patients with schizophrenia

Study	CX3CL1	CX3CR1	Comment
Bergon et al. [154]	NA	mRNA expression, decreased	Meta-analyses of postmortem brain and blood samples from patients with schizophrenia Brain regions included in the study: the prefrontal, frontal and temporal cortices, cerebellum, hippocampus, striatum and thalamus RT-qPCR examination of peripheral blood mononuclear cells from patients with schizophrenia
Fries et al. [155]	NA	mRNA expression, decreased	Genome-wide analysis of peripheral blood mononuclear cells from veterans with schizophrenia
Li et al. [156]	NA	mRNA expression, decreased	Datasets integrated analysis of samples from patients with schizophrenia Hippocampus
Gandal et al. [157]	Differential gene expression, decreased	Differential gene expression, decreased	Analyses of microarray gene expression data of postmortem samples from patients with schizophrenia Frontal and parietal cortex
[158]	Differential gene expression, decreased; variously expressed isoforms	Differential gene expression, decreased; variously expressed isoforms	Analyses of RNA-sequencing data of postmortem samples from patients with schizophrenia Frontal and temporal cortex
Ishizuka et al. [159]	NA	Ala55Thr variant in *CX3CR1* gene	Destabilization of the receptor gene’s conformation leading to the increased risk of schizophrenia
Ormel et al. [160]	NA	mRNA expression, increased in one of the phenotypes within the cells	Monocyte-derived microglia-like cells obtained from peripheral blood mononuclear cells of patients with schizophrenia
Zhang et al. [161]	NA	mRNA expression	Postmortem samples from patients with schizophrenia
		Unchanged (patients with schizophrenia versus controls)	Dorsal lateral prefrontal cortex, anterior cingulate cortex
		Increased (suicide completers with schizophrenia versus non-suicide subjects affected by the condition)	Anterior cingulate cortex
Hill et al. [162]	mRNA expression, unchanged	mRNA expression, unchanged	Postmortem samples from patients with schizophrenia
			Orbitofrontal cortex
	Protein level, decreased	Protein level, unchanged	Dorsolateral prefrontal cortex

*NA* not assessed

### Experimental data

One of the approaches to investigate schizophrenia-like disturbances in animals involves the maternal immune activation (MIA) paradigm [163, 164] (Table 2). Most often, MIA is generated by the administration of immunostimulants, for example, lipopolysaccharide (LPS) [165–169] or polyinosinic:polycytidylic acid (Poly I:C) [170, 171] to pregnant females of rodents. Current evidence showed alterations in the CX3CL1–CX3CR1 system in male offspring of MIA-treated Wistar rat dams [172]. The changes were present already in the early life of animals when an increase in the hippocampal *Cx3cl1* expression and CX3CR1 level, as well as cortical CX3CL1 production, was observed in descendants prenatally exposed to LPS. At the same time, MIA with Poly I:C elevated CX3CL1 level in the frontal cortex and decreased CX3CR1 release in the hippocampus of young rats. The disturbances of the CX3CL1–CX3CR1 axis were accompanied by alterations in the expression of microglial markers and the profile of cytokines released in the brains of juveniles in both MIA models [172]. Along with these results, the MIA-subjected offspring displayed multiple behavioral schizophrenia-like disturbances (e.g., PPI deficits and an aggressive phenotype) in adulthood. These malfunctions depended on the immunostimulant used and were accompanied by a reduction in hippocampal and a raise in cortical CX3CL1 levels in LPS- and Poly I:C-exposed animals, respectively [172]. In another article from this research group [173], the expression of the *Cx3cl1–Cx3cr1* dyad was not affected, while the protein levels of CX3CL1 in the hippocampus and CX3CR1 in the frontal cortex were down-regulated after MIA with LPS in adult offspring without a deficit in PPI. The additional acute challenge with LPS later in life, according to the “two-hit” hypothesis of schizophrenia, decreased levels of hippocampal CX3CL1 in rats with altered PPI and cortical CX3CR1 in animals without such behavioral deficiency [173]. In similar experimental conditions, yet applying MIA with Poly I:C, the mRNA expression of both *Cx3cl1* and *Cx3cr1* was reduced in the hippocampus of adult descendants without PPI deficit [174]. Simultaneously, the protein levels of the CX3CL1–CX3CR1 pair were upregulated in the frontal cortex of these animals. The additional injection of Poly I:C in adulthood decreased cortical *Cx3cr1* expression and increased hippocampal CX3CL1 level in offspring without impairment in PPI [174]. The results came from the experiments on Sprague-Dawley rats, which suggests that the different strains exert notable effects on the outcome of examinations in MIA models. Abnormalities in CX3CR1 levels have been also measured in microglia isolated from brains of mice subjected to prenatal Poly I:C injection [175, 176]. Mattei et al. [175] presented the diminished mRNA expression of the receptor in these cells obtained from the hippocampus of male offspring. The alteration was accompanied by deficits in social behavior and PPI (in part of animals) as well as working memory impairment [175]. In contrast, flow cytometry revealed that the cells of female descendants prenatally challenged with MIA were characterized by a significantly bigger population of microglia expressing CX3CR1 [176]. The change did not persist until adulthood and preceded deficits in PPI as those were noted only in the later stage of a female’s life. As postulated by the authors, these observations may indicate intensified synaptic processes occurring in response to Poly I:C administration. Furthermore, MIA did not influence PPI and led to a lasting decrease in CX3CR1 level in male offspring as the reduction was detected both in adolescence and adulthood [176]. Hui et al. [177] showed data, where prenatal immune challenge with Poly I:C in mice resulted in partly sex-dependent behavioral schizophrenia-like disturbances (for instance increased repetitive behavior, anxiety, reduced sociability and deficits in PPI) but no disturbances in *Cx3cl1* and *Cx3cr1* gene expression in brains of offspring were identified. A significant, although preliminary observation in the context of schizophrenia-associated abnormalities implementing the MIA model with Poly I:C was also provided by Estes et al. [178] in their preprint article. In the frontal cortex of male offspring mice, the *Cx3cr1* expression was oscillating throughout development with a decrease in mRNA levels at birth and postnatal day 14 (P14) and an increase at P7 and P60. It showed that the expression of the receptor gene was particularly up-regulated at the beginning of synaptogenesis (P7) and declined during the peak of this process and spine formation (P14). These age-specific changes in the *Cx3cr1* transcript level implicate MIA-induced microglial dysfunctions that trigger alterations in cortical networks [178]. The evidence seems to support the reports on impaired anatomical and functional connectivity in the cerebral cortex of patients with schizophrenia [179–181]. Consistent results were described by Garré et al. [182]. MIA with Poly I:C in mice caused dendritic spine loss, impairments in learning-dependent dendritic spine formation and deficits in learning tasks which were mediated by CX3CR1-highly expressing monocytes via TNF-α-dependent mechanisms. Recently, Bordeleau et al. [183] reported a different approach in inducing MIA and showed that exposure to a high-fat diet resulted in maternal systemic inflammation and simultaneously decreased the mRNA expression of *Cx3cr1* in the hippocampus of male offspring mice.

**Table 2:** Summary of reports on the CX3CL1–CX3CR1 axis protein levels and mRNA expression in the studies using maternal immune activation paradigm with LPS or Poly I:C

MIA induction agent	Sex, strain and species	CX3CL1–CX3CR1 axis	Additional information	References
LPS	Male Wistar rats	↑ *Cx3cl1* (hippocampus), ≡ *Cx3cl1* (frontal cortex) and *Cx3cr1* (hippocampus, frontal cortex), ↑ CX3CL1 (frontal cortex) and CX3CR1 (hippocampus), ≡ CX3CL1 (hippocampus) and CX3CR1 (frontal cortex) in young offspring ↓ CX3CL1 (hippocampus), ≡ CX3CL1 (frontal cortex) and CX3CR1 (hippocampus, frontal cortex) in adult offspring	Alterations in the mRNA expression of microglial markers and the profile of cytokines released in the brains of young offspring Behavioral schizophrenia-like disturbances (e.g., PPI deficits and an aggressive phenotype) in adulthood	[172]
Poly I:C		≡ *Cx3cl1* and *Cx3cr1* (hippocampus, frontal cortex), ↑ CX3CL1 (frontal cortex), ≡ CX3CL1 (hippocampus), ↓ CX3CR1 (hippocampus), ≡ CX3CR1 (frontal cortex) in young offspring ↑ CX3CL1 (frontal cortex), ≡ CX3CL1 (hippocampus) and CX3CR1 (hippocampus, frontal cortex) in adult offspring		
LPS	Male Sprague–Dawley rats	≡ *Cx3cl1* and *Cx3cr1* (hippocampus, frontal cortex) in adult offspring ≡ CX3CL1 and CX3CR1 (hippocampus, frontal cortex) in adult offspring with a deficit in PPI ↓ CX3CL1 (frontal cortex) and CX3CR1 (hippocampus), ≡ CX3CL1 (hippocampus) and CX3CR1 (frontal cortex) in adult offspring without a deficit in PPI ↓ CX3CL1 (hippocampus) in offspring with PPI deficit after additional challenge with LPS in adulthood ↓ CX3CR1 (frontal cortex) in offspring without PPI deficit after additional challenge with LPS in adulthood	Behavioral schizophrenia-like changes (increased exploratory activity and anxiety-like behaviors) in adulthood Occurrence of two phenotypes in PPI (with and without deficit) Adult offspring were additionally exposed to the acute challenge with LPS in adulthood, according to the “two-hit” hypothesis of schizophrenia	[173]
Poly I:C	Male Sprague-Dawley rats	↓ *Cx3cl1* and *Cx3cr1* (hippocampus), ≡ *Cx3cl1* and *Cx3cr1* (frontal cortex) in adult offspring with a deficit in PPI ≡ *Cx3cl1* and *Cx3cr1* (hippocampus, frontal cortex) in adult offspring without a deficit in PPI ↓ *Cx3cr1* (frontal cortex) in adult offspring without PPI deficit after additional challenge with Poly I:C in adulthood ≡ CX3CL1 and CX3CR1 (hippocampus, frontal cortex) in adult offspring with a deficit in PPI ↑ CX3CL1 and CX3CR1 (frontal cortex), ≡ CX3CL1 and CX3CR1 (hippocampus) in adult offspring without a deficit in PPI ↓ *Cx3cr1* (frontal cortex) in offspring without PPI deficit after additional challenge with Poly I:C in adulthood ↑ CX3CL1 (hippocampus) in offspring without PPI deficit after additional challenge with Poly I:C in adulthood	Behavioral schizophrenia-like disturbances (diminished number of aggressive interactions, depressive-like episodes, increased exploratory activity) Occurrence of two phenotypes in PPI (with and without deficit) Adult offspring were additionally exposed to the acute challenge with Poly I:C in adulthood, according to the “two-hit” hypothesis of schizophrenia	[174]
Poly I:C	Male C57BL/6 mice	↓ *Cx3cr1* (microglial cells isolated from the hippocampus) in adult offspring	Deficits in social behavior and PPI (in part of animals) as well as working memory impairment	[175]
Poly I:C	Female and male BALB/c mice	↑ Population of microglia (isolated from complete brains) expressing CX3CR1 in young female offspring that did not persist until adulthood ↓ CX3CR1 level in male offspring both in adolescence and adulthood	Deficits in PPI only in adult female offspring	[176]
Poly I:C	Female and male C57BL/6 mice	≡ *Cx3cl1* and *Cx3cr1* (ventromedial prefrontal cortex, hippocampal dentate gyrus and cerebellum)	Partly sex-dependent behavioral schizophrenia-like disturbances (for instance increased repetitive behavior, anxiety, reduced sociability and deficits in PPI)	[177]
Poly I:C	Male C57BL/6 mice	Age-specific changes in *Cx3cr1* expression: ↓ at birth and P14, ↑ at P7 and P60	*Cx3cr1* expression was particularly up-regulated at the beginning of synaptogenesis (P7) and declined during the peak of this process and spine formation (P14)	[178]
Poly I:C	Female and male chimeric and transgenic mice	CX3CR1-highly expressing monocytes	Dendritic spine loss, impairments in learning-dependent dendritic spine formation and deficits in learning tasks	[182]
High-fat diet	Female and male C57BL/6N	↓ *Cx3cr1* (hippocampus) in adolescent male offspring ≡ *Cx3cr1* (hippocampus) in adolescent female offspring	Similar phenotypes in both sexes for IL-6-driven immune priming and microglial morphology Sex-dependent changes in transcriptomic and astrocyte-microglia interaction	[183]

↑ (increased), ↓ (decreased), ≡ (unchanged)

Further evidence regarding the involvement of the CX3CL1–CX3CR1 pair in the pathogenesis of schizophrenia has been contributed by multiple studies on genetic models with a knockout of the receptor gene. Zhou et al. [184] applied a social isolation model of the disease in *Cx3cr1*-deficient mice and examined the schizophrenia-related behaviors. Unlike control animals exposed to the procedure, the knockouts did not display deficits in PPI. Moreover, the CX3CR1 level was up-regulated in the medial prefrontal cortex, hippocampus and nucleus accumbens of the isolated wild-type mice, suggesting that the receptor might participate in the examined schizophrenia-like behaviors [184]. Another feature observed for *Cx3cr1*−/− animals was a transient reduction of microglia during the early postnatal period that resulted in impaired synaptic pruning [185]. The authors stated that the lack of the receptor gene caused a decrease in synaptic transmission, attenuation of functional brain connectivity, intensified repetitive behavior and deficits in social interaction. Squarzoni et al. [186] suggested that a mild shift in neocortical positioning of microglia depicted in mice lacking the *Cx3cr1* gene could contribute to defects in postnatal synaptogenesis and cortical networks. Besides, *Cx3cr1* knockouts exhibited reduced baseline connectivity from the prefrontal cortex to the dorsal hippocampus during the habituation phase in the social interaction test [187]. This report seems to be particularly relevant in the context of data in the suffering from schizophrenia for whom sorely impaired connectivity between the hippocampus and the prefrontal cortex was shown [188, 189].

The current study by Lebovitz et al. [190] revealed that antibiotics-driven maternal microbiome dysbiosis (MMD), which is considered a model of neurodevelopmental disorders including schizophrenia, led to social impairments in male offspring. The deficiency coincided with an increased protein level of CX3CR1 in the prefrontal cortex of those mice. The application of *Cx3cr1*^GFP/GFP^ knockout animals allowed to demonstrate that MMD-reared descendants developed the changes in behavior due to dysfunction of the CX3CL1–CX3CR1 signaling and disrupted synaptic modeling. Notably, the presence of a gut commensal bacterium strain, *Lactobacillus murinus* HU-1, was sufficient to prevent social alterations and microglial activation in MMD-affected offspring [190]. Experiments in a pharmacological model of schizophrenia-like cognitive deficits induced by repeated ketamine administration showed the effect of cannabidiol (CBD) on the *Cx3cr1* transcript level [191]. As presented by the researchers, the CBD treatment caused the up-regulation of the receptor expression in the prefrontal cortex of male offspring of Sprague-Dawley rats. Therefore, the evidence implies that the CX3CL1–CX3CR1 axis might be crucial in the disease course and could provide a new target for future therapy.

## Conclusions

The literature data from reports in patients concerning the role of the CX3CL1–CX3CR1 axis in the pathogenesis of schizophrenia remain inconsistent and, thus, difficult to unambiguously interpret. More information has been provided by the studies in animal models of the disease (e.g., implementing MIA); however, those are often confounded with the discrepancies in experimental conditions, including species or strains of animals, a protocol of immunostimulant administration or even paradigm of behavioral examinations. Nevertheless, all of the observations shed a light and increasingly implicate the involvement of CX3CR1 and its ligand in mechanisms underlying schizophrenia. To date, the particular interest in the CX3CL1–CX3CR1 system seems to indirectly result from its extensive role in maintaining the homeostasis of processes in the CNS that are often indicated as disturbed in the course of that disorder (Fig. 2). Yet, further research is needed to a profound understanding of the exact contribution of this signaling pathway in schizophrenia.

## References

[1] Rössler W, Joachim Salize H, Van Os J, Riecher-Rössler A (2005). Size of burden of schizophrenia and psychotic disorders. Eur Neuropsychopharmacol.

[2] Chong HY, Teoh SL, Wu DBC, Kotirum S, Chiou CF, Chaiyakunapruk N (2016). Global economic burden of schizophrenia: a systematic review. Neuropsychiatr Dis Treat.

[3] Charlson FJ, Ferrari AJ, Santomauro DF, Diminic S, Stockings E, Scott JG (2018). Global epidemiology and burden of schizophrenia: findings from the global burden of disease study 2016. Schizophr Bull.

[4] Jaaro-Peled H, Sawa A (2020). Neurodevelopmental factors in schizophrenia. Psychiatr Clin N Am.

[5] Silveira C, Marques-Teixeira J, De Bastos-Leite AJ (2012). More than one century of schizophrenia: an evolving perspective. J Nerv Ment Dis.

[6] Tandon R, Gaebel W, Barch DM, Bustillo J, Gur RE, Heckers S (2013). Definition and description of schizophrenia in the DSM-5. Schizophr Res.

[7] Kahn RS, Sommer IE, Murray RM, Meyer-Lindenberg A, Weinberger DR, Cannon TD (2015). Schizophrenia. Nat Rev Dis Primers.

[8] Salleh MR (2004). The genetics of schizophrenia. Malays J Med Sci.

[9] Foley C, Corvin A, Nakagome S (2017). Genetics of schizophrenia: ready to translate?. Curr Psychiatry Rep.

[10] Trifu SC, Kohn B, Vlasie A, Patrichi B-E (2020). Genetics of schizophrenia. Exp Ther Med.

[11] Wheeler AL, Voineskos AN (2014). A review of structural neuroimaging in schizophrenia: from connectivity to connectomics. Front Hum Neurosci.

[12] Díaz-Soto CM, Castaño-Pérez GA, Pineda-Salazar DA (2020). Cannabis, schizophrenia and cognition: the contribution of brain connectivity. Adicciones.

[13] Adhikari BM, Hong LE, Sampath H, Chiappelli J, Jahanshad N, Thompson PM (2019). Functional network connectivity impairments and core cognitive deficits in schizophrenia. Hum Brain Mapp.

[14] Hummer TA, Yung MG, Goñi J, Conroy SK, Francis MM, Mehdiyoun NF (2020). Functional network connectivity in early-stage schizophrenia. Schizophr Res.

[15] Yao B, Neggers SFW, Kahn RS, Thakkar KN (2020). Altered thalamocortical structural connectivity in persons with schizophrenia and healthy siblings. NeuroImage Clin.

[16] Brisch R, Saniotis A, Wolf R, Bielau H, Bernstein HG, Steiner J (2014). The role of dopamine in schizophrenia from a neurobiological and evolutionary perspective: old fashioned, but still in vogue. Front Psychiatry.

[17] Selvaraj S, Arnone D, Cappai A, Howes O (2014). Alterations in the serotonin system in schizophrenia: a systematic review and meta-analysis of postmortem and molecular imaging studies. Neurosci Biobehav Rev.

[18] de Jonge JC, Vinkers CH, Hulshoff Pol HE, Marsman A (2017). GABAergic mechanisms in schizophrenia: linking postmortem and In vivo studies. Front Psychiatry.

[19] Uno Y, Coyle JT (2019). Glutamate hypothesis in schizophrenia. Psychiatry Clin Neurosci.

[20] Egerton A, Grace AA, Stone J, Bossong MG, Sand M, McGuire P (2020). Glutamate in schizophrenia: neurodevelopmental perspectives and drug development. Schizophr Res.

[21] Mäki-Marttunen V, Andreassen OA, Espeseth T (2020). The role of norepinephrine in the pathophysiology of schizophrenia. Neurosci Biobehav Rev.

[22] Stilo SA, Murray RM (2019). Non-genetic factors in schizophrenia. Curr Psychiatry Rep.

[23] Rokita KI, Dauvermann MR, Mothersill D, Holleran L, Holland J, Costello L (2021). Childhood trauma, parental bonding, and social cognition in patients with schizophrenia and healthy adults. J Clin Psychol.

[24] Lipner E, Murphy SK, Ellman LM (2019). Prenatal maternal stress and the cascade of risk to schizophrenia spectrum disorders in offspring. Curr Psychiatry Rep.

[25] Canetta SE, Brown AS (2012). Prenatal infection, maternal immune activation, and risk for schizophrenia. Transl Neurosci.

[26] Kneeland RE, Fatemi SH (2013). Viral infection, inflammation and schizophrenia. Prog Neuro-Psychopharmacol Biol Psychiatry.

[27] Cordeiro CN, Tsimis M, Burd I (2015). Infections and brain development. Obstet Gynecol Surv.

[28] Costas-Carrera A, Garcia-Rizo C, Bitanihirwe B, Penadés R (2020). Obstetric complications and brain imaging in schizophrenia: a systematic review. Biol Psychiatry Cogn Neurosci Neuroimaging.

[29] He P, Chen G, Guo C, Wen X, Song X, Zheng X (2018). Long-term effect of prenatal exposure to malnutrition on risk of schizophrenia in adulthood: evidence from the Chinese famine of 1959–1961. Eur Psychiatry.

[30] Ayhan Y, McFarland R, Pletnikov MV (2016). Animal models of gene–environment interaction in schizophrenia: a dimensional perspective. Prog Neurobiol.

[31] Moran P, Stokes J, Marr J, Bock G, Desbonnet L, Waddington J (2016). Gene × environment interactions in schizophrenia: evidence from genetic mouse models. Neural Plast.

[32] Bioque M, Mas S, Costanzo MC, Cabrera B, Lobo A, González-Pinto A (2019). Gene-environment interaction between an endocannabinoid system genetic polymorphism and cannabis use in first episode of psychosis. Eur Neuropsychopharmacol.

[33] Karl T, Arnold JC (2014). Schizophrenia: a consequence of gene-environment interactions?. Front Behav Neurosci.

[34] Smigielski L, Jagannath V, Rössler W, Walitza S, Grünblatt E (2020). Epigenetic mechanisms in schizophrenia and other psychotic disorders: a systematic review of empirical human findings. Mol Psychiatry.

[35] Cromby J, Chung E, Papadopoulos D, Talbot C (2019). Reviewing the epigenetics of schizophrenia. J Ment Health.

[36] Richetto J, Meyer U (2021). Epigenetic modifications in schizophrenia and related disorders: molecular scars of environmental exposures and source of phenotypic variability. Biol Psychiatry.

[37] Stuart MJ, Baune BT (2014). Chemokines and chemokine receptors in mood disorders, schizophrenia, and cognitive impairment: a systematic review of biomarker studies. Neurosci Biobehav Rev.

[38] Reale M, Costantini E, Greig NH (2021). Cytokine imbalance in schizophrenia. From research to clinic: potential implications for treatment. Front Psychiatry.

[39] Barichello T, Simoes LR, Quevedo J, Zhang XY (2020). Microglial activation and psychotic disorders: evidence from pre-clinical and clinical studies. Curr Top Behav Neurosci.

[40] Comer AL, Carrier M, Tremblay MÈ, Cruz-Martín A (2020). The inflamed brain in schizophrenia: the convergence of genetic and environmental risk factors that lead to uncontrolled neuroinflammation. Front Cell Neurosci.

[41] Misiak B, Bartoli F, Carrà G, Stańczykiewicz B, Gładka A, Frydecka D (2021). Immune-inflammatory markers and psychosis risk: a systematic review and meta-analysis. Psychoneuroendocrinology.

[42] Fond G, Lançon C, Korchia T, Auquier P, Boyer L (2020). The role of inflammation in the treatment of schizophrenia. Front Psychiatry.

[43] Leonard BE (2005). Is there an immunologic basis for schizophrenia?. Expert Rev Clin Immunol.

[44] Miller BJ, Bickley P, Seabolt W, Mellor A, Kirkpatrick B (2011). Meta-analysis of cytokine alterations in schizophrenia: clinical status and antipsychotic effects. Biol Psychiatry.

[45] Reale M, Patruno A, De Lutiis MA, Pesce M, Felaco M, Di Giannantonio M (2011). Dysregulation of chemo-cytokine production in schizophrenic patients versus healthy controls. BMC Neurosci.

[46] Gallego JA, Blanco EA, Husain-Krautter S, Madeline Fagen E, Moreno-Merino P, del Ojo-Jiménez JA (2018). Cytokines in cerebrospinal fluid of patients with schizophrenia spectrum disorders: new data and an updated meta-analysis. Schizophr Res.

[47] De Witte L, Tomasik J, Schwarz E, Guest PC, Rahmoune H, Kahn RS (2014). Cytokine alterations in first-episode schizophrenia patients before and after antipsychotic treatment. Schizophr Res.

[48] Romeo B, Brunet-Lecomte M, Martelli C, Benyamina A (2018). Kinetics of cytokine levels during antipsychotic treatment in schizophrenia: a meta-analysis. Int J Neuropsychopharmacol.

[49] Paul-Samojedny M, Kowalczyk M, Suchanek R, Owczarek A, Fila-Danilow A, Szczygiel A (2010). Functional polymorphism in the interleukin-6 and interleukin-10 genes in patients with paranoid schizophrenia—a case-control study. J Mol Neurosci.

[50] Paul-Samojedny M, Owczarek A, Kowalczyk M, Suchanek R, Palacz M, Kucia K (2013). Association of interleukin 2 (IL-2), interleukin 6 (IL-6), and TNF-alpha (TNFα) gene polymorphisms with paranoid schizophrenia in a Polish population. J Neuropsychiatry Clin Neurosci.

[51] Suchanek-Raif R, Raif P, Kowalczyk M, Paul-Samojedny M, Kucia K, Merk W (2018). Promoter polymorphisms of TNF-α gene as a risk factor for schizophrenia. Arch Med Res.

[52] Monji A, Kato T, Kanba S (2009). Cytokines and schizophrenia: microglia hypothesis of schizophrenia. Psychiatry Clin Neurosci.

[53] Aguzzi A, Barres BA, Bennett ML (2013). Microglia: scapegoat, saboteur, or something else?. Science.

[54] Bazan JF, Bacon KB, Hardiman G, Wang W, Soo K, Rossi D (1997). A new class of membrane-bound chemokine with a CX3Cmotif. Nature.

[55] Pan Y, Lloyd C, Zhou H, Dolich S, Deeds J, Gonzalo J-A (1997). Neurotactin, a membrane-anchored chemokine upregulated in brain inflammation. Nature.

[56] Garton KJ, Gough PJ, Blobel CP, Murphy G, Greaves DR, Dempsey PJ (2001). Tumor necrosis factor-alpha-converting enzyme (ADAM17) mediates the cleavage and shedding of fractalkine (CX3CL1). J Biol Chem.

[57] Zieger M, Ahnelt PK, Uhrin P (2014). CX3CL1 (Fractalkine) protein expression in normal and degenerating mouse retina: in vivo studies. PLoS ONE.

[58] Mackay CR (1997). Chemokines: what chemokine is that?. Curr Biol.

[59] Ostuni MA, Guellec J, Hermand P, Durand P, Combadiere C, Pincet F (2014). CX3CL1, a chemokine finely tuned to adhesion: critical roles of the stalk glycosylation and the membrane domain. Biol Open.

[60] Hughes PM, Botham MS, Frentzel S, Mir A, Perry VH (2002). Expression of fractalkine (CX3CL1) and its receptor, CX3CR1, during acute and chronic inflammation in the rodent CNS. Glia.

[61] Winter AN, Subbarayan MS, Grimmig B, Weesner JA, Moss L, Peters M (2020). Two forms of CX3CL1 display differential activity and rescue cognitive deficits in CX3CL1 knockout mice. J Neuroinflamm.

[62] Hundhausen C, Misztela D, Berkhout TA, Broadway N, Saftig P, Reiss K (2003). The disintegrin-like metalloproteinase ADAM10 is involved in constitutive cleavage of CX3CL1 (fractalkine) and regulates CX3CL1-mediated cell-cell adhesion. Blood.

[63] Tsou CL, Haskell CA, Charo IF (2001). Tumor necrosis factor-α-converting enzyme mediates the inducible cleavage of fractalkine. J Biol Chem.

[64] Bourd-Boittin K, Basset L, Bonnier D, L’Helgoualc’h A, Samson M, Théret N (2009). CX3CL1/fractalkine shedding by human hepatic stellate cells: contribution to chronic inflammation in the liver. J Cell Mol Med.

[65] Uchida M, Ito T, Nakamura T, Igarashi H, Oono T, Fujimori N (2013). ERK pathway and sheddases play an essential role in ethanol-induced CX3CL1 release in pancreatic stellate cells. Lab Investig.

[66] Clark AK, Yip PK, Malcangio M (2009). The liberation of fractalkine in the dorsal horn requires microglial cathepsin S. J Neurosci.

[67] Jones BA, Riegsecker S, Rahman A, Beamer M, Aboualaiwi W, Khuder SA (2013). Role of ADAM-17, p38 MAPK, Cathepsins, and the proteasome pathway in the synthesis and shedding of fractalkine/CX3CL1 in rheumatoid arthritis. Arthritis Rheum.

[68] Fonović UP, Jevnikar Z, Kos J (2013). Cathepsin S generates soluble CX3CL1 (fractalkine) in vascular smooth muscle cells. Biol Chem.

[69] Hundhausen C, Schulte A, Schulz B, Andrzejewski MG, Schwarz N, von Hundelshausen P (2007). Regulated shedding of transmembrane chemokines by the disintegrin and metalloproteinase 10 facilitates detachment of adherent leukocytes. J Immunol.

[70] Peraire J, Vidal F, Plana M, Domingo P, Coll B, Viladés C (2007). Polymorphisms in the 3′ untranslated region of the fractalkine (CX3CL1) gene and the risk of HIV-1 infection and disease progression. AIDS.

[71] Ma G, Yang J, Zhao B, Huang C, Wang R (2019). Correlation between CCL2, CALCA, and CX3CL1 gene polymorphisms and chronic pain after cesarean section in Chinese Han women. Medicine (Baltimore).

[72] Jin SG, Chen GL, Yang SL, Zhao MY (2015). Gene-gene interactions among CX3CL1, LEPR and IL-6 related to coronary artery disease in Chinese Han population. Int J Clin Exp Pathol.

[73] Zhang X, Feng X, Cai W, Liu T, Liang Z, Sun Y (2015). Chemokine CX3CL1 and its receptor CX3CR1 are associated with human atherosclerotic lesion volnerability. Thromb Res.

[74] Debette S, Bevan S, Dartigues JF, Sitzer M, Lorenz M, Ducimetière P (2009). Fractalkine receptor/ligand genetic variants and carotid intima-media thickness. Stroke.

[75] Peng Q, Shi L, Kong Y, Yan Y, Zhan J, Wen Y (2020). CX3CL1 rs170364 gene polymorphism has a protective effect against major depression by enhancing its transcriptional activity. Brain Res.

[76] Cardona AE, Sasse ME, Liu L, Cardona SM, Mizutani M, Savarin C (2008). Scavenging roles of chemokine receptors: chemokine receptor deficiency is associated with increased levels of ligand in circulation and tissues. Blood.

[77] Tarozzo G, Bortolazzi S, Crochemore C, Chen SC, Lira AS, Abrams JS (2003). Fractalkine protein localization and gene expression in mouse brain. J Neurosci Res.

[78] Harrison JK, Jiang Y, Chen S, Xia Y, Maciejewski D, McNamara RK (1998). Role for neuronally derived fractalkine in mediating interactions between neurons and CX3CR1-expressing microglia. Proc Natl Acad Sci USA.

[79] Papadopoulos EJ, Sassetti C, Saeki H, Yamada N, Kawamura T, Fitzhugh DJ (1999). Fractalkine, a CX3C chemokine, is expressed by dendritic cells and is up-regulated upon dendritic cell maturation. Eur J Immunol.

[80] Muehlhoefer A, Saubermann LJ, Gu X, Luedtke-Heckenkamp K, Xavier R, Blumberg RS (2000). Fractalkine is an epithelial and endothelial cell-derived chemoattractant for intraepithelial lymphocytes in the small intestinal mucosa. J Immunol.

[81] Lucas AD, Chadwick N, Warren BF, Jewell DP, Gordon S, Powrie F (2001). The transmembrane form of the CX3CL1 chemokine fractalkine is expressed predominantly by epithelial cells in vivo. Am J Pathol.

[82] Hou SM, Hou CH, Liu JF (2017). CX3CL1 promotes MMP-3 production via the CX3CR1, c-Raf, MEK, ERK, and NF-ΚB signaling pathway in osteoarthritis synovial fibroblasts. Arthritis Res Ther.

[83] Greaves DR, Häkkinen T, Lucas AD, Liddiard K, Jones E, Quinn CM (2001). Linked chromosome 16q13 chemokines, macrophage-derived chemokine, fractalkine, and thymus- and activation-regulated chemokine, are expressed in human atherosclerotic lesions. Arterioscler Thromb Vasc Biol.

[84] Ludwig A, Berkhout T, Moores K, Groot P, Chapman G (2002). Fractalkine is expressed by smooth muscle cells in response to IFN-γ and TNF-α and is modulated by metalloproteinase activity. J Immunol.

[85] Hatori K, Nagai A, Heisel R, Ryu JK, Kim SU (2002). Fractalkine and fractalkine receptors in human neurons and glial cells. J Neurosci Res.

[86] O’Sullivan SA, Gasparini F, Mir AK, Dev KK (2016). Fractalkine shedding is mediated by p38 and the ADAM10 protease under pro-inflammatory conditions in human astrocytes. J Neuroinflamm.

[87] Sowa JE, Ślusarczyk J, Trojan E, Chamera K, Leśkiewicz M, Regulska M (2017). Prenatal stress affects viability, activation, and chemokine signaling in astroglial cultures. J Neuroimmunol.

[88] Yoshida H, Imaizumi T, Fujimoto K, Matsuo N, Kimura K, Cui XF (2001). Synergistic stimulation, by tumor necrosis factor-α and interferon-γ, of fractalkine expression in human astrocytes. Neurosci Lett.

[89] Harrison JK, Barber CM, Lynch KR (1994). cDNA cloning of a G-protein-coupled receptor expressed in rat spinal cord and brain related to chemokine receptors. Neurosci Lett.

[90] Raport CJ, Schweickart VL, Eddy RL, Shows TB, Gray PW (1995). The orphan G-protein-coupled receptor-encoding gene V28 is closely related to genes for chemokine receptors and is expressed in lymphoid and neural tissues. Gene.

[91] Imai T, Hieshima K, Haskell C, Baba M, Nagira M, Nishimura M (1997). Identification and molecular characterization of fractalkine receptor CX 3 CR1, which mediates both leukocyte migration and adhesion. Cell.

[92] Raucci R, Costantini S, Castello G, Colonna G (2014). An overview of the sequence features of N- and C-terminal segments of the human chemokine receptors. Cytokine.

[93] Darbandi-Tehrani K, Hermand P, Carvalho S, Dorgham K, Couvineau A, Lacapère J (2010). Subtle conformational changes between CX3CR1 genetic variants as revealed by resonance energy transfer assays. FASEB J.

[94] Zhang R, Wang LY, Wang YF, Wu CR, Lei CL, Wang MX (2015). Associations between the T280M and V249I snps in CX3CR1 and the risk of age-related macular degeneration. Investig Ophthalmol Vis Sci.

[95] Faure S, Meyer L, Costagliola D, Vaneensberghe C, Genin E, Autran B (2000). Rapid progression to AIDS in HIV+ individuals with a structural variant of the chemokine receptor CX3CR1. Science.

[96] Lopez-Lopez A, Gamez J, Syriani E, Morales M, Salvado M, Rodríguez MJ (2014). CX3CR1 is a modifying gene of survival and progression in amyotrophic lateral sclerosis. PLoS ONE.

[97] Calvo A, Moglia C, Canosa A, Cammarosano S, Ilardi A, Bertuzzo D (2018). Common polymorphisms of chemokine (C-X3-C motif) receptor 1 gene modify amyotrophic lateral sclerosis outcome: a population-based study. Muscle Nerve.

[98] Moatti D, Faure S, Fumeron F, El Walid AM, Seknadji P, McDermott DH (2001). Polymorphism in the fractalkine receptor CX3CR1 as a genetic risk factor for coronary artery disease. Blood.

[99] Brand S, Hofbauer K, Dambacher J, Schnitzler F, Staudinger T, Pfennig S (2006). Increased expression of the chemokine fractalkine in Crohn’s disease and association of the fractalkine receptor T280M polymorphism with a fibrostenosing disease phenotype. Am J Gastroenterol.

[100] Arli B, Irkec C, Menevse S, Yilmaz A, Alp E (2013). Fractalkine gene receptor polymorphism in patients with multiple sclerosis. Int J Neurosci.

[101] Sirois-Gagnon D, Chamberland A, Perron S, Brisson D, Gaudet D, Laprise C (2011). Association of common polymorphisms in the fractalkine receptor (CX3CR1) with obesity. Obesity.

[102] Sakai M, Takeuchi H, Yu Z, Kikuchi Y, Ono C, Takahashi Y (2018). Polymorphisms in the microglial marker molecule CX3CR1 affect the blood volume of the human brain. Psychiatry Clin Neurosci.

[103] Lee S, Latha K, Manyam G, Yang Y, Rao A, Rao G (2020). Role of CX3CR1 signaling in malignant transformation of gliomas. Neuro Oncol.

[104] Maciejewski-Lenoir D, Chen S, Feng L, Maki R, Bacon KB (1999). Characterization of fractalkine in rat brain cells: migratory and activation signals for CX3CR-1-expressing microglia. J Immunol.

[105] Wolf Y, Yona S, Kim KW, Jung S (2013). Microglia, seen from the CX3CR1 angle. Front Cell Neurosci.

[106] Choi JY, Kim JH, Hossain FMA, Uyangaa E, Park SO, Kim B (2019). Indispensable role of CX3CR1+ dendritic cells in regulation of virus-induced neuroinflammation through rapid development of antiviral immunity in peripheral lymphoid tissues. Front Immunol.

[107] Juremalm M, Nilsson G (2005). Chemokine receptor expression by mast cells. Chem Immunol Allergy.

[108] Combadiere C, Salzwedel K, Smith ED, Tiffany HL, Berger EA, Murphy PM (1998). Identification of CX3CR1. A chemotactic receptor for the human CX3C chemokine fractalkine and a fusion coreceptor for HIV-1. J Biol Chem.

[109] Geissmann F, Jung S, Littman DR (2003). Blood monocytes consist of two principal subsets with distinct migratory properties. Immunity.

[110] Al-Aoukaty A, Rolstad B, Giaid A, Maghazachi AA (1998). MIP-3α, MIP-3β and fractalkine induce the locomotion and the mobilization of intracellular calcium, and activate the heterotrimeric G proteins in human natural killer cells. Immunology.

[111] Schulz C, Schäfer A, Stolla M, Kerstan S, Lorenz M, Von Brühl ML (2007). Chemokine fractalkine mediates leukocyte recruitment to inflammatory endothelial cells in flowing whole blood: a critical role for P-selectin expressed on activated platelets. Circulation.

[112] Gan AM, Butoi ED, Manea A, Simion V, Stan D, Parvulescu MM (2013). Inflammatory effects of resistin on human smooth muscle cells: up-regulation of fractalkine and its receptor, CX3CR1 expression by TLR4 and Gi-protein pathways. Cell Tissue Res.

[113] Boddeke EWGM, Meigel I, Frentzel S, Biber K, Renn LQ, Gebicke-Härter P (1999). Functional expression of the fractalkine (CX3C) receptor and its regulation by lipopolysaccharide in rat microglia. Eur J Pharmacol.

[114] Wang A, Yang T, Zhang L, Jia L, Wu Q, Yao S (2018). IP3-mediated calcium signaling is involved in the mechanism of fractalkine-induced hyperalgesia response. Med Sci Monit.

[115] Montagud-Romero S, Montesinos J, Pavón FJ, Blanco-Gandia MC, Ballestín R, Rodríguez de Fonseca F (2020). Social defeat-induced increase in the conditioned rewarding effects of cocaine: role of CX3CL1. Prog Neuro-Psychopharmacol Biol Psychiatry.

[116] Castro-Sánchez S, García-Yagüe ÁJ, Kügler S, Lastres-Becker I (2019). CX3CR1-deficient microglia shows impaired signalling of the transcription factor NRF2: implications in tauopathies. Redox Biol.

[117] Butoi ED, Gan AM, Manduteanu I, Stan D, Calin M, Pirvulescu M (2011). Cross talk between smooth muscle cells and monocytes/activated monocytes via CX3CL1/CX3CR1 axis augments expression of pro-atherogenic molecules. Biochim Biophys Acta Mol Cell Res.

[118] Galán-Ganga M, García-Yagüe ÁJ, Lastres-Becker I (2019). Role of MSK1 in the induction of NF-κB by the chemokine CX3CL1 in microglial cells. Cell Mol Neurobiol.

[119] Huang LY, Ma BW, Ma JW, Wang F (2017). Fractalkine/CX3CR1 axis modulated the development of pancreatic ductal adenocarcinoma via JAK/STAT signaling pathway. Biochem Biophys Res Commun.

[120] Lee SJ, Namkoong S, Kim YM, Kim CK, Lee H, Ha KS (2006). Fractalkine stimulates angiogenesis by activating the Raf-1/MEK/ERK- and PI3K/Akt/eNOS-dependent signal pathways. Am J Physiol Heart Circ Physiol.

[121] Klosowska K, Volin MV, Huynh N, Chong KK, Halloran MM, Woods JM (2009). Fractalkine functions as a chemoattractant for osteoarthritis synovial fibroblasts and stimulates phosphorylation of mitogen-activated protein kinases and Akt. Clin Exp Immunol.

[122] Gu HJ, Zuo S, Liu HY, Gu LL, Yang XW, Liao J (2019). CX3CR1 participates in pulmonary angiogenesis in experimental hepatopulmonary syndrome mice through inhibiting AKT/ERK signaling pathway and regulating NO/NOS Release. Eur Rev Med Pharmacol Sci.

[123] Zhang Y, Zheng J, Zhou Z, Zhou H, Wang Y, Gong Z (2015). Fractalkine promotes chemotaxis of bone marrow-derived mesenchymal stem cells towards ischemic brain lesions through Jak2 signaling and cytoskeletal reorganization. FEBS J.

[124] Huang LY, Chen P, Xu LX, Zhou YF, Zhang YP, Yuan YZ (2012). Fractalkine upregulates inflammation through CX3CR1 and the Jak–Stat pathway in severe acute pancreatitis rat model. Inflammation.

[125] Davis CN, Harrison JK (2006). Proline 326 in the C terminus of murine CX3CR1 prevents G-protein and phosphatidylinositol 3-kinase-dependent stimulation of akt and extracellular signal-regulated kinase in Chinese hamster ovary cells. J Pharmacol Exp Ther.

[126] Liu P, Liang Y, Jiang L, Wang H, Wang S, Dong J (2018). CX3CL1/fractalkine enhances prostate cancer spinal metastasis by activating the Src/FAK pathway. Int J Oncol.

[127] Liang Y, Yi L, Liu P, Jiang L, Wang H, Hu A (2018). CX3CL1 involves in breast cancer metastasizing to the spine via the Src/FAK signaling pathway. J Cancer.

[128] Sheridan GK, Murphy KJ (2013). Neuron-glia crosstalk in health and disease: Fractalkine and CX3CR1 take centre stage. Open Biol.

[129] Paolicelli RC, Bisht K, Tremblay MÈ (2014). Fractalkine regulation of microglial physiology and consequences on the brain and behavior. Front Cell Neurosci.

[130] Arnoux I, Audinat E (2015). Fractalkine signaling and microglia functions in the developing brain. Neural Plast.

[131] Lauro C, Catalano M, Trettel F, Limatola C (2015). Fractalkine in the nervous system: neuroprotective or neurotoxic molecule?. Ann N Y Acad Sci.

[132] Ransohoff RM, El Khoury J (2016). Microglia in health and disease. Cold Spring Harb Perspect Biol.

[133] Chamera K, Trojan E, Szuster-Głuszczak M, Basta-Kaim A (2020). The potential role of dysfunctions in neuron-microglia communication in the pathogenesis of brain disorders. Curr Neuropharmacol.

[134] Lyons A, Lynch AM, Downer EJ, Hanley R, O’Sullivan JB, Smith A (2009). Fractalkine-induced activation of the phosphatidylinositol-3 kinase pathway attentuates microglial activation in vivo and in vitro. J Neurochem.

[135] Mattison HA, Nie H, Gao H, Zhou H, Hong JS, Zhang J (2013). Suppressed pro-inflammatory response of microglia in CX3CR1 knockout mice. J Neuroimmunol.

[136] White GE, McNeill E, Channon KM, Greaves DR (2014). Fractalkine promotes human monocyte survival via a reduction in oxidative stress. Arterioscler Thromb Vasc Biol.

[137] Li C, He J, Zhong X, Gan H, Xia Y (2018). CX3CL1/CX3CR1 axis contributes to angiotensin II-induced vascular smooth muscle cell proliferation and inflammatory cytokine production. Inflammation.

[138] Momtazmanesh S, Zare-Shahabadi A, Rezaei N (2019). Cytokine alterations in schizophrenia: an updated review. Front Psychiatry.

[139] Mongan D, Ramesar M, Föcking M, Cannon M, Cotter D (2020). Role of inflammation in the pathogenesis of schizophrenia: a review of the evidence, proposed mechanisms and implications for treatment. Early Interv Psychiatry.

[140] Paolicelli RC, Bolasco G, Pagani F, Maggi L, Scianni M, Panzanelli P (2011). Synaptic pruning by microglia is necessary for normal brain development. Science.

[141] Rogers JT, Morganti JM, Bachstetter AD, Hudson CE, Peters MM, Grimmig BA (2011). CX3CR1 deficiency leads to impairment of hippocampal cognitive function and synaptic plasticity. J Neurosci.

[142] Bolós M, Perea JR, Terreros-Roncal J, Pallas-Bazarra N, Jurado-Arjona J, Ávila J (2018). Absence of microglial CX3CR1 impairs the synaptic integration of adult-born hippocampal granule neurons. Brain Behav Immun.

[143] Hoshiko M, Arnoux I, Avignone E, Yamamoto N, Audinat E (2012). Deficiency of the microglial receptor CX3CR1 impairs postnatal functional development of thalamocortical synapses in the barrel cortex. J Neurosci.

[144] Hayashi-Takagi A (2017). Synapse pathology and translational applications for schizophrenia. Neurosci Res.

[145] Lima Caldeira G, Peça J, Carvalho AL (2019). New insights on synaptic dysfunction in neuropsychiatric disorders. Curr Opin Neurobiol.

[146] Neves G, Cooke SF, Bliss TVP (2008). Synaptic plasticity, memory and the hippocampus: a neural network approach to causality. Nat Rev Neurosci.

[147] Stoneham ET, Sanders EM, Sanyal M, Dumas TC (2010). Rules of engagement: factors that regulate activity-dependent synaptic plasticity during neural network development. Biol Bull.

[148] Bassi MS, Iezzi E, Gilio L, Centonze D, Buttari F (2019). Synaptic plasticity shapes brain connectivity: implications for network topology. Int J Mol Sci.

[149] Benes FM (2000). Emerging principles of altered neural circuitry in schizophrenia. Brain Res Rev.

[150] Floresco SB, Zhang Y, Enomoto T (2009). Neural circuits subserving behavioral flexibility and their relevance to schizophrenia. Behav Brain Res.

[151] Lewis DA, Sweet RA (2009). Schizophrenia from a neural circuitry perspective: advancing toward rational pharmacological therapies. J Clin Invest.

[152] Eisenberg DP, Berman KF (2010). Executive function, neural circuitry, and genetic mechanisms in schizophrenia. Neuropsychopharmacology.

[153] Cordon I, Nicolás MJ, Arrieta S, Lopetegui E, López-Azcárate J, Alegre M (2015). Coupling in the cortico-basal ganglia circuit is aberrant in the ketamine model of schizophrenia. Eur Neuropsychopharmacol.

[154] Bergon A, Belzeaux R, Comte M, Pelletier F, Hervé M, Gardiner EJ (2015). CX3CR1 is dysregulated in blood and brain from schizophrenia patients. Schizophr Res.

[155] Fries GR, Dimitrov DH, Lee S, Braida N, Yantis J, Honaker C (2018). Genome-wide expression in veterans with schizophrenia further validates the immune hypothesis for schizophrenia. Schizophr Res.

[156] Li WX, Dai SX, Liu JQ, Wang Q, Li GH, Huang JF (2016). Integrated analysis of Alzheimer’s disease and schizophrenia dataset revealed different expression pattern in learning and memory. J Alzheimer’s Dis.

[157] Gandal MJ, Haney JR, Parikshak NN, Leppa V, Ramaswami G, Hartl C (2018). Shared molecular neuropathology across major psychiatric disorders parallels polygenic overlap. Science.

[158] Gandal MJ, Zhang P, Hadjimichael E, Walker RL, Chen C, Liu S (2018). Transcriptome-wide isoform-level dysregulation in ASD, schizophrenia, and bipolar disorder. Science.

[159] Ishizuka K, Fujita Y, Kawabata T, Kimura H, Iwayama Y, Inada T (2017). Rare genetic variants in CX3CR1 and their contribution to the increased risk of schizophrenia and autism spectrum disorders. Transl Psychiatry.

[160] Ormel PR, Böttcher C, Gigase FAJ, Missall RD, van Zuiden W, Fernández Zapata MC (2020). A characterization of the molecular phenotype and inflammatory response of schizophrenia patient-derived microglia-like cells. Brain Behav Immun.

[161] Zhang L, Verwer RWH, Lucassen PJ, Huitinga I, Swaab DF (2020). Prefrontal cortex alterations in glia gene expression in schizophrenia with and without suicide. J Psychiatr Res.

[162] Hill SL, Shao L, Beasley CL (2020). Diminished levels of the chemokine fractalkine in post-mortem prefrontal cortex in schizophrenia but not bipolar disorder. World J Biol Psychiatry.

[163] Estes ML, McAllister AK (2016). Maternal immune activation: implications for neuropsychiatric disorders. Science.

[164] Brown AS, Meyer U (2018). Maternal immune activation and neuropsychiatric illness: a translational research perspective. Am J Psychiatry.

[165] Basta-Kaim A, Budziszewska B, Leśkiewicz M, Fijał K, Regulska M, Kubera M (2011). Hyperactivity of the hypothalamus-pituitary-adrenal axis in lipopolysaccharide-induced neurodevelopmental model of schizophrenia in rats: effects of antipsychotic drugs. Eur J Pharmacol.

[166] Basta-Kaim A, Fijał K, Budziszewska B, Regulska M, Leśkiewicz M, Kubera M (2011). Prenatal lipopolysaccharide treatment enhances MK-801-induced psychotomimetic effects in rats. Pharmacol Biochem Behav.

[167] Basta-Kaim A, Szczęsny E, Leśkiewicz M, Głombik K, Budziszewska B, Regulska M (2012). Maternal immune activation leads to age-related behavioral and immunological changes in male rat offspring—the effect of antipsychotic drugs. Pharmacol Rep.

[168] Basta-Kaim A, Fijał K, Ślusarczyk J, Trojan E, Głombik K, Budziszewska B (2015). Prenatal administration of lipopolysaccharide induces sex-dependent changes in glutamic acid decarboxylase and parvalbumin in the adult rat brain. Neuroscience.

[169] Wischhof L, Irrsack E, Osorio C, Koch M (2015). Prenatal LPS-exposure—a neurodevelopmental rat model of schizophrenia—differentially affects cognitive functions, myelination and parvalbumin expression in male and female offspring. Prog Neuro-Psychopharmacol Biol Psychiatry.

[170] Meyer U, Feldon J (2012). To poly(I:C) or not to poly(I:C): advancing preclinical schizophrenia research through the use of prenatal immune activation models. Neuropharmacology.

[171] Reisinger S, Khan D, Kong E, Berger A, Pollak A, Pollak DD (2015). The poly(I:C)-induced maternal immune activation model in preclinical neuropsychiatric drug discovery. Pharmacol Ther.

[172] Chamera K, Kotarska K, Szuster-Głuszczak M, Trojan E, Skórkowska A, Pomierny B (2020). The prenatal challenge with lipopolysaccharide and polyinosinic:polycytidylic acid disrupts CX3CL1-CX3CR1 and CD200-CD200R signalling in the brains of male rat offspring: a link to schizophrenia-like behaviours. J Neuroinflamm.

[173] Chamera K, Szuster-Głuszczak M, Trojan E, Basta-Kaim A (2020). Maternal immune activation sensitizes male offspring rats to lipopolysaccharide-induced microglial deficits involving the dysfunction of CD200-CD200R and CX3CL1-CX3CR1 systems. Cells.

[174] Chamera K, Trojan E, Kotarska K, Szuster-Głuszczak M, Bryniarska N, Tylek K (2021). Role of polyinosinic: polycytidylic acid-induced maternal immune activation and subsequent immune challenge in the behaviour and microglial cell trajectory in adult offspring: a study of the neurodevelopmental model of schizophrenia. Int J Mol Sci.

[175] Mattei D, Ivanov A, Ferrai C, Jordan P, Guneykaya D, Buonfiglioli A (2017). Maternal immune activation results in complex microglial transcriptome signature in the adult offspring that is reversed by minocycline treatment. Transl Psychiatry.

[176] Eßlinger M, Wachholz S, Manitz MP, Plümper J, Sommer R, Juckel G (2016). Schizophrenia associated sensory gating deficits develop after adolescent microglia activation. Brain Behav Immun.

[177] Hui CW, St-Pierre A, El Hajj H, Remy Y, Hébert SS, Luheshi GN (2018). Prenatal immune challenge in mice leads to partly sex-dependent behavioral, microglial, and molecular abnormalities associated with schizophrenia. Front Mol Neurosci.

[178] Estes ML, Elmer BM, Carter CC, McAllister AK. Maternal immune activation causes age-specific changes in cytokine receptor expression in offspring throughout development. BioRxiv. 2018;490466. 10.1101/490466.

[179] Zhou Y, Fan L, Qiu C, Jiang T. Prefrontal cortex and the dysconnectivity hypothesis of schizophrenia. Neurosci Bull. 2015;31:207–19. 10.1007/s12264-014-1502-8. 10.1007/s12264-014-1502-8 PMC5563697 25761914

[180] Pokorny VJ, Espensen-Sturges TD, Burton PC, Sponheim SR, Olman CA (2020). Aberrant cortical connectivity during ambiguous object recognition is associated with schizophrenia. Biol Psychiatry Cogn Neurosci Neuroimaging.

[181] Vittala A, Murphy N, Maheshwari A, Krishnan V (2020). Understanding cortical dysfunction in schizophrenia with TMS/EEG. Front Neurosci.

[182] Garré JM, Silva HM, Lafaille JJ, Yang G (2017). CX3CR1+ monocytes modulate learning and learning-dependent dendritic spine remodeling via TNF-α. Nat Med.

[183] Bordeleau M, Lacabanne C, de Cossío LF, Vernoux N, Savage J, González-Ibáñez F (2020). Microglial and peripheral immune priming is partially sexually dimorphic in adolescent mouse offspring exposed to maternal high-fat diet. J Neuroinflamm.

[184] Zhou H, Wang J, Zhang Y, Shao F, Wang W (2020). The role of microglial CX3CR1 in schizophrenia-related behaviors induced by social isolation. Front Integr Neurosci.

[185] Zhan Y, Paolicelli RC, Sforazzini F, Weinhard L, Bolasco G, Pagani F (2014). Deficient neuron-microglia signaling results in impaired functional brain connectivity and social behavior. Nat Neurosci.

[186] Squarzoni P, Oller G, Hoeffel G, Pont-Lezica L, Rostaing P, Low D (2014). Microglia modulate wiring of the embryonic forebrain. Cell Rep.

[187] Zhan Y (2015). Theta frequency prefrontal-hippocampal driving relationship during free exploration in mice. Neuroscience.

[188] Meyer-Lindenberg AS, Olsen RK, Kohn PD, Brown T, Egan MF, Weinberger DR (2005). Regionally specific disturbance of dorsolateral prefrontal-hippocampal functional connectivity in schizophrenia. Arch Gen Psychiatry.

[189] Bähner F, Meyer-Lindenberg A (2017). Hippocampal–prefrontal connectivity as a translational phenotype for schizophrenia. Eur Neuropsychopharmacol.

[190] Lebovitz Y, Kowalski EA, Wang X, Kelly C, Lee M, McDonald V (2019). Lactobacillus rescues postnatal neurobehavioral and microglial dysfunction in a model of maternal microbiome dysbiosis. Brain Behav Immun.

[191] Kozela E, Krawczyk M, Kos T, Juknat A, Vogel Z, Popik P (2020). Cannabidiol improves cognitive impairment and reverses cortical transcriptional changes induced by ketamine, in schizophrenia-like model in rats. Mol Neurobiol.

